# Sex and age differences in COVID-19 mortality in Europe

**DOI:** 10.21203/rs.3.rs-61444/v1

**Published:** 2020-08-19

**Authors:** Linda Juel Ahrenfeldt, Martina Otavova, Kaare Christensen, Rune Lindahl-Jacobsen

**Affiliations:** University of Southern Denmark; University of Southern Denmark; University of Southern Denmark; University of Southern Denmark

**Keywords:** COVID-19, SARS-CoV-2, sex difference, mortality, Europe

## Abstract

**Aim:**

To examine the magnitude of sex differences in survival from the Coronavirus Disease 2019 (COVID-19) in Europe across age and countries. We hypothesise that men have higher mortality than women at any given age, but that sex differences will decrease with age as only the strongest men survive to older ages.

**Methods:**

We used population data from Institut National D’Études Demographiques on cumulative deaths due to COVID-19 from February to June 2020 in 10 European countries: Denmark, Norway, Sweden, The Netherlands, England & Wales, France, Germany, Italy, Spain and Portugal. For each country, we calculated cumulative mortality rates stratified by age and sex and corresponding relative risks for men vs. women.

**Results:**

The relative risk of dying from COVID-19 was higher for men than for women in almost all age groups in all countries. The overall relative risk ranged from 1.11 (95% CI 1.01–1.23) in Portugal to 1.54 (95% CI 1.49–1.58) in France. In most countries, sex differences increased until ages 60–69 years, but decreased thereafter with the smallest sex difference at ages 80+.

**Conclusions:**

Despite variability in data collection and time coverage among countries, we illustrate an overall similar pattern of sex differences in COVID-19 mortality in Europe.

## Introduction

Despite the fact that women suffer greater morbidity than men, particularly late in life [1], women outlive men in almost all countries of the world [2]. This is the case even under the harshest conditions where mortality is very high, e.g. during severe famines and epidemics [3]. Since December 2019, the emergence of the coronavirus disease 2019 (COVID-19) has been reported in Wuhan, China, with infection outbreaks across China and around the world [4, 5]. Data from early reported cases in China suggest that the mortality rate from COVID-19 is higher for infected men than for infected women [6–8] with fatality rates of 2.8% in men vs. 1.7% in women [8]. A recent study from mainland China found that the case fatality rate (CFR) (the risk of dying among persons diagnosed with COVID-19) was lower in female patients (4.0%) than in male patients (7.2%); however, sex differences varied between regions and were age-dependent with significant differences among patients aged 30 years and above [9]. In agreement with this finding, the Global Health 50/50 research initiative, which presents an overview of sex-disaggregated data from countries worldwide, demonstrates that despite similar numbers of COVID-19 cases in men and women there is an increased CFR in men [10]. A review collecting epidemiological data on confirmed COVID-19 cases in Europe and China until April 1, 2020 found that the male to female COVID-19 CFRs reported from France, Italy, Spain, Germany, Switzerland and China were relatively homogenous and ranged between 1.7 and 1.8 [11]. In addition, pooled data from Italy, Spain, Germany and Switzerland comprising 227,219 confirmed cases and 14,364 deaths suggested that, although the male to female CFR was consistently elevated through all age groups, the sex difference was most pronounced at middle age, particularly at ages 50–59 years [11]. Correspondingly, data on COVID-19 cases and deaths obtained from outbreaks in China, Italy and the New York City demonstrated a higher mortality for men than for women at all ages, with the scale of difference between sexes being consistent with that found for more common causes of mortality, such as heart disease [12].

Here we examine the magnitude of sex differences in survival from COVID-19 across age groups and countries in Europe. This will allow us to investigate the consistency of sex differences across regions and thus to map the epidemiology of the disease. However, due to the highly variable rate of COVID-19 by age, also the age-specific mortality from COVID-19 are considered. We hypothesise that men have higher mortality than women at any given age, but that sex differences in mortality from COVID-19 will decrease with age as only the strongest men survive to older ages [1].

## Material And Methods

### Study popula tion

This study was based on 10 European countries (Denmark, Norway, Sweden, The Netherlands, England & Wales, France, Germany, Italy, Spain and Portugal) that reported daily cumulative deaths due to COVID-19 by sex and age available on the Institut National D’Etudes Demographiques (INED) website [13]. The data collection methods and the time period covered differed for each country with the earliest date of February 14, 2020 and the latest date of June 29, 2020 (Please see Supplementary Table 1). Sex- and age-specific population sizes (numbers) for the 10 European countries can be found in Supplementary Table 2.

### Statistical analysis

Sex-specific cumulative mortality rates (CMR) from COVID-19 per 100,000 men and women were calculated for each day for the covered time periods in each country. In addition, CMRs and relative risks (RRs) with 95% confidence intervals (CIs) were calculated in each country in four age groups (<60, 60–69, 70–79 and 80+ years) using data from the last day covered by the study. The Wald test was used to investigate differences between the RRs of adjacent age groups. R version 3.5.0 and Stata version 16 were used for the analyses.

## Results

In all European countries, the CMRs increased with advancing age (Table 1). The highest CMRs were found in England & Wales with 91.0 (95% CI 89.9–92.1) per 100,000 men and 72.4 (95% CI 71.4–73.3) per 100.000 women. High CMRs were also found in two Southern European countries, Italy (66.2, 95% CI 65.3–67.2 per 100,000 men and 45.4, 95% CI 44.7–46.2 per 100,000 women) and Spain (50.3, 95% CI 49.4–51.2 per 100,000 men and 37.2, 95% CI 36.4–38.0 per 100,000 women). The countries with the lowest mortality from COVID-19 were Norway (5.0, 95% CI 4.2–5.9 per 100,000 men and 4.3, 95% CI 3.6–5.2 per 100.000 women), Denmark (11.8, 95% CI 10.6–13.2 per 100,000 men and 8.9, 95% CI 7.9–10.0 per 100,000 women), and Germany (12.1, 95% CI 11.8–12.5 per 100,000 men and 9.5, 95% CI 9.2–9.8 per 100,000 women) (Table 1). The patterns for the CMRs between February and June 2020 revealed an overall similar trend for men and women; however, higher CMRs were found for men than for women, particularly in Western and Southern Europe (Supplementary Figure 1).

When investigating the RRs for men vs. women, we found higher mortality among men in all age groups except from ages <60 years for Denmark and Norway and ages 80+ years for Norway (Table 1). The overall RRs ranged from 1.11 (95% CI 1.01–1.23) in Portugal to 1.54 (95% CI 1.49, 1.58) in France; however, the overall RR in Norway was non-significant (RR = 1.15, 95% CI 0.89–1.47). In most countries, sex differences increased from ages <60 years to 60–69 years but decreased thereafter (Table 1). Although not all differences in RRs between adjacent age groups were significant, differences in RRs between the oldest age groups were significant in all countries (Table 1). The largest sex differences were found at ages 60–69 years in Norway, England & Wales, Germany and Italy, up to age 69 years in Sweden, up to age 79 years in the Netherlands, France and Portugal, from 60–79 years in Spain and at ages 70–79 years in Denmark. The smallest sex difference was found at ages 80+ years in all countries (Table 1). Supplementary Figure 2 illustrates the RRs for men vs. women in the four age groups across Europe. We found the largest sex difference in Norway (RR = 4.27, 95% CI 0.92–19.76), Sweden (RR = 3.03, 95% CI 2.17–4.24), and Italy (RR = 2.91, 95% CI 2.59–3.26) for people below age 60 years, in Norway (RR = 5.97, 95% CI 1.76–20.28), Italy (RR = 3.49, 95% CI 3.23–3.78) and Germany (RR = 2.99, 95% CI 2.57–3.49) for people aged 60–69 years, in Denmark (RR = 2.77, 95% CI 1.99–3.86), Italy (RR = 2.71, 95% CI 2.59–2.83) and France (RR = 2.64, 95% CI 2.47–2.81) for people aged 70–79 years and in France (RR = 1.92, 95% CI 1.85–1.99), Spain (RR = 1.65, 95% CI 1.60–1.70) and Italy (RR = 1.59, 95% CI 1.55–1.63) for people aged 80+ years (Supplementary Figure 2 and Table 1). Overall, the largest sex differences in COVID-19 mortality were found in France, Italy and Spain.

## Discussion

By using population estimates from the INED website (February to June 2020) on daily cumulative deaths by age and sex due to COVID-19 in 10 European countries, we found that the risk of death increased with age, and that men had higher mortality from COVID-19 than women in almost all age groups across the 10 European countries. In most countries, sex differences increased from ages <60 years to 60–69 years but decreased thereafter with the smallest sex difference at ages 80+ years in all countries.

Generally, there is a male disadvantage in mortality rates with women living longer than men in almost all countries of the world [2]. A recent study investigating the survival in seven populations under extreme conditions from famines, epidemics and slavery found that even when mortality is very high, women survived on average better than men [3]. Although the biggest contribution to these differentials came from the large mortality differences among infants, the authors found that for all populations, the extreme age, defined as the age to which 5% of the population survived, was higher for women than for men, supporting the hypothesis of an overall ability of females to withstand higher-mortality crises better than males [3]. The study supports that the survival advantage of women has fundamental biological underpinnings, but also that the female advantage is modulated by a complex interaction of biological, environmental and social factors [3].

The higher mortality from COVID-19 for men than for women was overall similar to that found in other coronaviruses during the last two decades, such as the severe respiratory syndrome coronavirus (SARSCoV) and the Middle East respiratory syndrome (MERS-CoV) [14–17]. The reasons for the sex differences in COVID-19 are likely multifactorial and include differences in immune response, biological differences between the sexes, differences in lifestyle such as smoking habits as well as differences in underlying comorbidities [18–20]. Although recent evidence suggests that European women overall have slightly more comorbidities than European men [21], men are generally reported to have more life-threatening conditions, such as cardiovascular diseases, whereas women tend to have more non-fatal chronic diseases, such as migraine, musculoskeletal and autoimmune diseases as well as physical limitations [22–25]. Thus, the male disadvantage in risk of death from COVID-19 may to some extent be explained by the relatively higher prevalence of underlying comorbidities such as cardiovascular disease, hypertension, diabetes and chronic lung disease for men [10]. According to a recent study, the scale of difference between sexes in COVID-19 mortality is consistent with that found for heart disease, but greater than that seen for death due to diabetes, or combined influenza and pneumonia [12].

Although we demonstrated a higher mortality from COVID-19 for men than for women in almost all age groups, we found, as hypothesised, a reduction in the relative risk of mortality for men at later ages, consistently with findings elsewhere [12]. A narrowing of the sex gap with increasing age may be consistent with a survival effect, which leaves the healthiest men in the sample [1]. However, if oestrogen protects women from the most serious complications of COVID-19, women may be most protected before the menopause due to the higher serum oestrogen levels [26].

Evidence from studies investigating sex differences in COVID-19 mortality stresses the importance of addressing the impact of sex differences on disease epidemics, outbreaks, and pandemics in public health policies and efforts. All countries should report data separately by sex, and research studies should, whenever possible, analyse the interactions between age and sex in COVID-19 morbidity and mortality [11].

The strength of this study was the ability to analyse sex differences in COVID-19 mortality by age groups in 10 European countries showing an overall similar pattern of sex differences, despite the variability in data collection and time coverage among countries. Not all countries have reported data separately by sex, and this study was limited to the European countries providing sex-disaggregated data. Another limitation was that the frequency, recording and reporting of COVID-19 deaths differ from one country to another, but may also differ within countries. The cause of death can be certified by different biological tests, by clinical diagnosis, and by mentioning the infection on the death certificates [13]. Therefore, a cross-national comparison of results should be done with caution.

By using population data on daily cumulative deaths due to COVID-19 from 10 European countries, we confirm a consistently higher mortality from COVID-19 among European men than among European women in almost all age groups. In most countries, sex differences increased from ages <60 years to 60–69 years but decreased thereafter, with the smallest sex difference at ages 80+ years in all countries. This study highlights the importance of addressing the impact of sex on mortality from disease epidemics, but studies using individual-level data are needed to confirm an interaction between age and sex in COVID-19 mortality in order to guide clinical care personal and to address questions of whether men require additional surveillance, prevention, and earlier intervention than women.

## Supplementary Material

Supplement

Supplement

Supplement

## Figures and Tables

**Table 1 -:** Cumulative mortality rates (CMRs) of COVID-19 per 100,000 men and women and relative risks (RRs) for men vs. women with 95% confidence intervals (CIs) over age groups by 10 European countries

	CMR	95% CI	CMR	95% CI	RR	95% CI
**Denmark**						
**Overall**	11.84	(10.64–13.18)	8.92	(7.88–10.01)	1.33	(1.13–1.56)
**<60**	0.55	(0.96–0.99)	0.23	(0.08–0.58)	2.33	(0.82–6.61)
**60–69**	11.34	(8.10–1.58)	6.52	(4.19–10.10)	1.74	(1.03–2.95)
**70–79**	45.88	(38.29–54.94)	16.54	(12.36–22.05)	2.77	(1.99–3.86)*
**80+**	157.62	(135.28–183.55)	112.92	(97.50–13.07)	1.40	(1.13–1.72)*
**Norway**						
**Overall**	4.95	(4.17–5.88)	4.32	(3.58–5.21)	1.15	(0.89–1.47)
**<60**	0.43	(0.21–0.84)	0.10	(0.02–0.40)	4.27	(0.92–19.76)
**60–69**	6.17	(3.77–9.96)	1.03	(0.27–3.29)	5.97	(1.76–20.28)
**70–79**	18.04	(12.94–25.03)	8.44	(5.23–13.45)	2.14	(1.23–3.71)*
**80+**	76.30	(59.81–97.16)	64.87	(52.53–80.03)	1.18	(0.86–1.61)*
**Sweden**						
**Overall**	52.27	(50.33–54.29)	46.11	(44.27–48.01)	1.13	(1.07–1.20)
**<60**	3.65	(3.09–4.31)	1.20	(0.87–1.62)	3.03	(2.17–4.24)
**60–69**	40.22	(35.18–45.97)	16.53	(13.40–20.37)	2.43	(1.91–3.10)
**70–79**	142.07	(131.69–153.25)	76.42	(69.11–84.49)	1.86	(1.64–2.10)*
**80+**	773.56	(737.25–811.64)	573.95	(548.22–600.86)	1.35	(1.26–1.44)*
**The Netherlands**						
**Overall**	39.13	(37.83–40.48)	31.59	(30.43–32.80)	1.24	(1.18–1.30)
**<60**	1.98	(1.66–2.36)	0.97	(0.75–1.26)	2.04	(1.50–2.76)
**60–69**	31.98	(28.68–35.66)	15.78	(13.51–18.42)	2.03	(1.68–2.44)
**70–79**	142.95	(134.45–151.95)	74.26	(68.44–80.58)	1.92	(1.74–2.13)
**80+**	601.69	(574.82–629.79)	393.81	(376.57–411.82)	1.53	(1.43–1.62)*
**England & Wales**						
**Overall**	90.99	(89.90–92.09)	72.36	(71.40–73.32)	1.26	(1.23–1.28)
**<60**	9.00	(8.62–9.40)	5.06	(4.77–5.36)	1.78	(1.66–1.91)
**60–69**	100.84	(97.32–104.48)	50.04	(47.64–52.57)	2.01	(1.90–2.14)*
**70–79**	296.63	(289.67–303.75)	156.15	(151.39–161.07)	1.90	(1.82–1.97)*
**80+**	1234.8	(1214.91–1254.77)	848.04	(834.55–861.75)	1.46	(1.42–1.48)*
**France**						
**Overall**	34.81	(34.18–35.46)	22.65	(22.16–23.17)	1.54	(1.49–1.58)
**<60**	3.45	(3.22–3.69)	1.69	(1.54–1.86)	2.04	(1.81–2.29)
**60–69**	42.82	(40.78–44.97)	15.88	(14.71–18.65)	2.70	(2.46–2.95)*
**70–79**	114.51	(110.45–118.71)	43.42	(41.14–45.82)	2.64	(2.47–2.81)
**80+**	390.98	(381.06–401.15)	203.60	(19.82–20.91)	1.92	(1.85–1.99)*
**Germany**						
**Overall**	12.11	(11.77–12.45)	9.50	(9.21–9.80)	1.27	(1.22–1.33)
**<60**	1.01	(0.91–1.14)	0.40	(0.33–0.48)	2.55	(2.06–3.16)
**60–69**	12.63	(11.67–13.67)	4.21	(3.69–4.81)	2.99	(2.57–3.49)*
**70–79**	38.79	(36.77–40.92)	15.76	(14.59–17.02)	2.46	(2.42–2.70)*
**80+**	131.46	(126.53–136.57)	89.09	(85.94–92.35)	1.48	(1.40–1.55)*
**Italy**						
**Overall**	66.24	(65.31–67.17)	45.42	(44.68–46.18)	1.46	(1.43–1.49)
**<60**	5.37	(5.06–5.69)	1.85	(1.67–2.04)	2.91	(2.59–3.26)
**60–69**	73.80	(70.99–76.71)	21.12	(19.70–22.64)	3.49	(3.23–3.78)*
**70–79**	226.55	(220.96–232.28)	83.51	(80.41–86.73)	2.71	(2.59–2.83)*
**80+**	594.16	(582.36–606.20)	373.17	(365.98–380.49)	1.59	(1.55–1.63)*
**Spain**						
**Overall**	50.27	(49.37–51.20)	37.18	(36.41–37.96)	1.35	(1.32–1.39)
**<60**	3.76	(3.48–4.06)	1.74	(1.55–1.95)	2.17	(1.89–2.48)
**60–69**	50.41	(47.70–53.26)	19.72	(18.11–21.47)	2.56	(2.31–2.83)*
**70–79**	187.42	(181.13–193.93)	73.52	(69.95–77.28)	2.55	(2.40–2.70)
**80+**	597.80	(583.26–612.70)	362.22	(353.52–371.13)	1.65	(1.60–1.70)*
**Portugal**						
**Overall**	16.12	(15.02–17.30)	14.49	(13.50–15.55)	1.11	(1.01–1.23)
**<60**	1.30	(9.69–1.75)	0.66	(0.44–0.99)	1.96	(1.21–3.19)
**60–69**	16.29	(13.28–19.94)	6.65	(4.93–8.95)	2.45	(1.72–3.48)
**70–79**	43.76	(37.74–50.72)	21.87	(18.21–26.25)	2.00	(1.59–2.52)
**80+**	192.50	(175.44–211.20)	140.14	(129.22–151.98)	1.37	(1.22–1.55)*

* Significant differences in relative risks between the specific age group and the age group above.
