# Supplementary figures and images for: Sex and age differences in COVID-19 mortality in Europe
Source: Res Sq. 2020 Aug 19:rs.3.rs-61444. Preprint. [Version 1] doi: 10.21203/rs.3.rs-61444/v1 (PMC7444295; doi:10.21203/rs.3.rs-61444/v1)

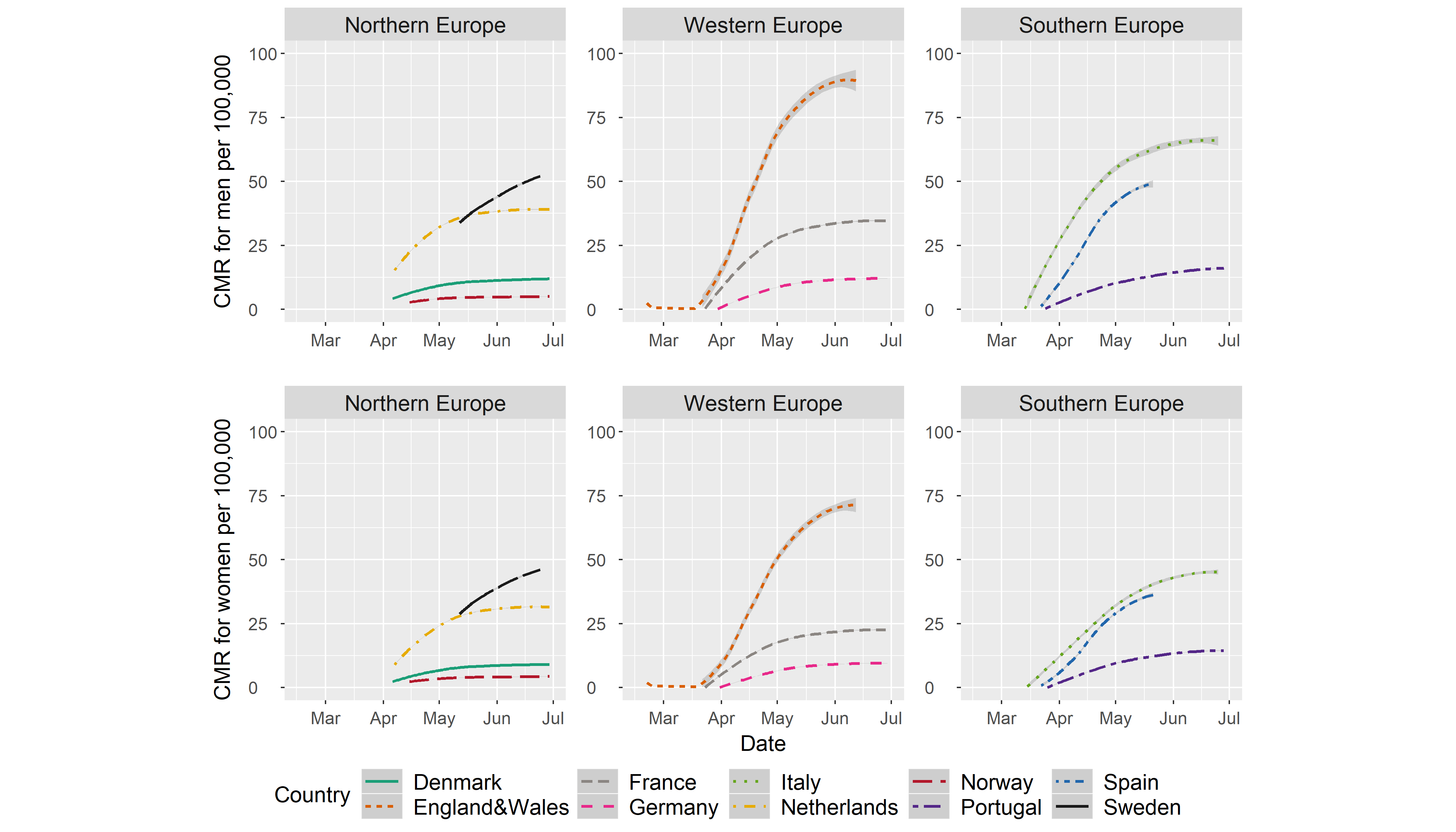

Supplement: Supplement [file SuppFig1.tiff]

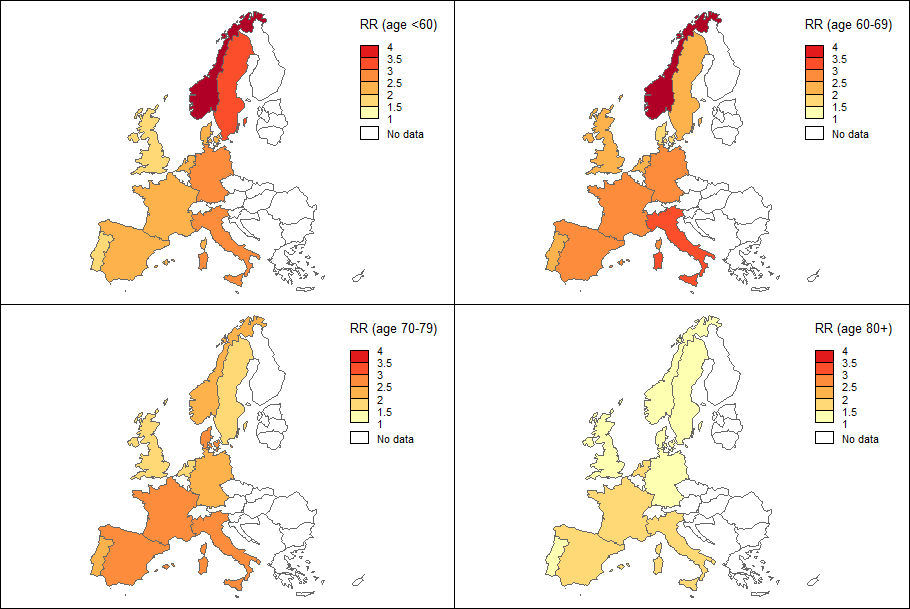

Supplement: Supplement [file SuppFig2.tiff]
