# Supplementary material for: Sex and age differences in COVID-19 mortality in Europe
Source: Res Sq. 2020 Aug 19:rs.3.rs-61444. Preprint. [Version 1] doi: 10.21203/rs.3.rs-61444/v1 (PMC7444295; doi:10.21203/rs.3.rs-61444/v1)
Supplement: Supplement [file SuppTables.docx]

Supplementary Tables

Supplementary Table 1 - Information on data source and time coverage for each of the 10 European countries included in the study

| Country | Data Source | Time Coverage |
| --- | --- | --- |
| Denmark | Source: Statens Serum Institut (SSI)  File content:   - Daily cumulative number of all COVID-19 tested deaths by sex, all places of death included - Daily cumulative number of all COVID-19 tested deaths reassigned on the date of occurrence (sex and age combined), all places of death included | 06-04-2020 –  29-06-2020 |
| Norway | Source: Norwegian Institute of Public Health (FHI)  File content:   - Daily cumulative number of all COVID-19 tested deaths, by age and sex, all places of death included - Daily cumulative number of all COVID-19 tested deaths, age and sex combined, by place of death (hospitals, other health institutions, home) | 15-04-2020 –  29-06-2020 |
| Sweden | Source: Public Health Agency of Sweden (PHAS)  Conseil national de la sante et du bien-entre (NBHW)  File content:   - Daily cumulative COVID-19 tested deaths, regardless of the cause of death, by 10-year age groups and by sex separately - Daily cumulative deaths where COVID-19 is the underlined cause of death on the death certificate, by 10-year age groups and by sex - Daily cumulative deaths where COVID-19 is the underlined cause of death of the death certificate, according to the place of death - Daily cumulative deaths age and sex combined, all places of deaths included, according to the date of occurrence of the death | 12-05-2020 –  24-06-2020 |
| The Netherlands | Source: Netherlands National Institute for Public Health and the Environment (RIVM)  File content:   - Daily cumulative number of deaths published by age and sex; all places of death included - Daily cumulative number of deaths reassigned on the date of occurrence (sex and age combined), all places of death are included | 07-04-2020 –  29-06-2020 |
| England & Wales | Source: Office for National Statistics (ONS)  National Health Service (NHS)  Public Health Wales (GIG)  File Content:   - Weekly cumulative number of all COVID-19 certified deaths, by age and sex, according to the date of publication of the death (corrected daily), all places of death in England and Wales - Daily cumulative deaths (mainly) occurred in hospitals and COVID-19 tested, by sex and age - Daily cumulative deaths (mainly) occurred in hospitals and COVID-19 tested, sex and age combined. Wales - Weekly cumulative number of COVID-19 certified deaths (sex and age combined), according to the place of death. Great Britain and Wales | 14-02-2020 –  12-06-2020 |
| France | Source: Public Health France (SpF)  File content:   - Daily cumulative deaths occurred in hospitals, published by age and sex - Daily cumulative deaths (age and sex combined) of individuals living in social & medical institutions. Distinction of deaths occurring in institutions and in hospitals (reporting on a platform – coverage not specified) | 22-03-2020 –  28-06-2020 |
| Germany | Source: Robert Koch-Institut (RKI)  File content:   - Daily cumulative deaths with COVID-19 laboratory diagnostic confirmation, published by age and sex, all places of deaths included - Daily cumulative deaths by age and sex combined, all places of deaths included | 30-03-2020 –  29-06-2020 |
| Italy | Source: Institut superieur de la sante (ISS)  Civil protection  File content:   - Daily cumulative COVID-19 tested deaths by age (sexes combined) all places of death, daily publication (report ISS) - Daily cumulative COVID-19 tested deaths by age and sex, all places of death, biweekly publication (report ISS) - Daily cumulative COVID-19 tested deaths, age and sex combined (report civil protection) | 12-03-2020 –  25-06-2020 |
| Spain | Source: Ministry of Health, Consumption and Social Welfare (MSCBS)  Carlos III Health Institute (ISCiii)  File content:   - Daily cumulative COVID-19 tested deaths, occurred in hospitals, by age and sex - Daily cumulative deaths reported to the National Network of Public Health Surveillance (RENAVE) protocol by age groups, all places of death included - Daily cumulative COVID-19 tested deaths, age and sex combined, and all places of death included | 22-03-2020 –  21-05-2020 |
| Portugal | Source: National Health Service and General Health Department of the Ministry of Health in Portugal (SNS and DGS)  File content:   - Daily cumulative number of all COVID-19 tested deaths, by age and sex, all places of death included - Daily cumulative number of all COVID-19 tested deaths, age and sex combined | 23-03-2020 –  28-06-2020 |

Supplementary Table 2 - Sex- and age-specific population sizes (numbers) for the ten European countries included in the study

| Country | Total population | Men | Women |
| --- | --- | --- | --- |
| Denmark |  |  |  |
| Overall | 5 822 763 | 2 896 918 | 2 925 845 |
| <60 | 4 322 401 | 2 193 956 | 2 128 445 |
| 60-79 | 663 646 | 326 395 | 337 251 |
| 70-79 | 564 390 | 268076 | 296 314 |
| 80+ | 272 326 | 108 491 | 163 835 |
| Norway |  |  |  |
| Overall | 5 367 580 | 2 706 562 | 2 661 018 |
| <60 | 4 118 541 | 2 113 619 | 2 004 922 |
| 60-69 | 582 496 | 291 860 | 290 635 |
| 70-79 | 435 834 | 210 649 | 225 185 |
| 80+ | 230 710 | 90 434 | 140 276 |
| Sweden |  |  |  |
| Overall | 10 327 553 | 5 195 794 | 5 131 759 |
| <60 | 7 693 816 | 3 949 316 | 3 744 500 |
| 60-69 | 1 108 431 | 551 949 | 556 482 |
| 70-79 | 989 008 | 478 645 | 510 363 |
| 80+ | 536 298 | 215 884 | 320 414 |
| The Netherlands |  |  |  |
| Overall | 17 282 163 | 8 581 086 | 8 701 077 |
| <60 | 12 871 320 | 6 504 777 | 6 366 543 |
| 60-69 | 2 089 913 | 1 038 005 | 1 051 908 |
| 70-79 | 1 522 110 | 730 336 | 791 774 |
| 80+ | 798 820 | 307 968 | 490 852 |
| England & Wales |  |  |  |
| Overall | 59 115 809 | 29 215 251 | 29 900 558 |
| <60 | 45 050 537 | 22 680 711 | 22 369 826 |
| 60-69 | 6 240 802 | 3 041 536 | 3 199 239 |
| 70-79 | 4 885 277 | 2 308 296 | 2 576 981 |
| 80+ | 2 939 193 | 1 184 681 | 1 754 512 |
| France |  |  |  |
| Overall | 67 063 703 | 32 397 179 | 34 666 524 |
| <60 | 49 213 463 | 24 514 764 | 24 698 699 |
| 60-69 | 7 999 606 | 3 792 182 | 4 207 424 |
| 70-79 | 5 693 660 | 2 598 072 | 3 095 588 |
| 80+ | 4 156 974 | 1 492 161 | 2 664 813 |
| Germany |  |  |  |
| Overall | 83 019 213 | 40 966 691 | 42 052 522 |
| <60 | 59 641 767 | 30 450 818 | 29 190 949 |
| 60-69 | 10 302 411 | 4 987 359 | 5 315 052 |
| 70-79 | 7 685 929 | 3 503 497 | 4 182 432 |
| 80+ | 5 389 106 | 2 025 017 | 3 364 089 |
| Italy |  |  |  |
| Overall | 60 359 546 | 29 384 766 | 30 974 780 |
| <60 | 42 729 729 | 21 541 448 | 21 188 281 |
| 60-69 | 7 337 210 | 3 511 037 | 3 826 173 |
| 70-79 | 5 962 533 | 2 727 000 | 3 235 533 |
| 80+ | 4 330 074 | 1 605 281 | 2 724 793 |
| Spain |  |  |  |
| Overall | 47 100 395 | 23 089 389 | 24 011 006 |
| <60 | 35 057 016 | 17 713 808 | 17 343 208 |
| 60-69 | 5 281 877 | 2 543 236 | 2 738 641 |
| 70-79 | 3 900 550 | 1 771 960 | 2 128 590 |
| 80+ | 2 860 952 | 1 060 385 | 1 800 567 |
| Portugal |  |  |  |
| Overall | 10 276 617 | 4 852 366 | 5 424 251 |
| <60 | 7 363 638 | 3 604 196 | 3 759 442 |
| 60-69 | 128 6927 | 595 393 | 691 534 |
| 70-79 | 964 596 | 415 892 | 548 704 |
| 80+ | 661 456 | 236 885 | 424 571 |
